# Activity of Bambara Groundnut Seed Coat Extract Against *Shewanella* Species: Efficacy and Mechanisms of Action

**DOI:** 10.3390/foods13213516

**Published:** 2024-11-04

**Authors:** Suriya Palamae, Watcharapol Suyapoh, Onpreeya Boonrat, Bin Zhang, Muhamad Amin, Jirayu Buatong, Soottawat Benjakul

**Affiliations:** 1 International Center of Excellence in Seafood Science and Innovation, Faculty of Agro-Industry, Prince of Songkla University, Hat Yai 90110, Songkhla, Thailand; suriya.pal@psu.ac.th (S.P.); soottawat.b@psu.ac.th (S.B.); 2 Veterinary Pathology Unit, Department of Veterinary Science, Faculty of Veterinary Science, Prince of Songkla University, Hat Yai 90110, Songkhla, Thailand; watcharapol_su@hotmail.com; 3 Medical Science Research and Innovation Institute, Research and Development Office, Prince of Songkla University, Hat Yai 90110, Songkhla, Thailand; onpreeya.bo@psu.ac.th; 4 Key Laboratory of Health Risk Factors for Seafood of Zhejiang Province, College of Food Science and Pharmacy, Zhejiang Ocean University, Zhoushan 316022, China; zhangbin_ouc@163.com; 5 Department of Aquaculture, Faculty of Fisheries and Marine, Universitas Airlangga, Campus C Jl, Mulyorejo, Surabaya 60115, East Java, Indonesia; muhamad.amin@fpk.unair.ac.id; 6 Department of Food and Nutrition, Kyung Hee University, Seoul 02447, Republic of Korea

**Keywords:** *Shewanella* species, seed coat, Bambara groundnut, antimicrobial activity, natural preservative, active compounds

## Abstract

The Bambara groundnut is the indigenous legume in the southern part of Thailand. It contains a seed coat rich in polyphenols, which can serve as natural antimicrobial agents. The extracts from red and white seed coats of Bambara groundnuts, namely RSC and WSC, respectively, were prepared using an ultrasound-assisted extraction process. The extraction yield, total phenolic content (TPC), and antimicrobial activity of both extracts were examined. The RSC extract demonstrated a significantly higher extraction yield (8.35%) than WSC extract (2.34%) (*p* < 0.05). Furthermore, the TPC of RSC extract (420.98 ± 0.27 mg of gallic acid/g dry extract) was higher than that of WSC extract (28.29 ± 0.91 mg of gallic acid/g dry extract). The RSC extract exhibited stronger inhibition against *Shewanella putrefaciens* and *S. algae* than its WSC counterpart. Liquid chromatography quadrupole time-of-flight mass spectrometry (LC-Q-TOF/MS) analysis indicated that the RSC extract was rich in flavonoids and polyphenols, while the WSC extract contained more triterpenoid saponins. Time–kill kinetics showed that the RSC extract reduced bacterial loads in a dose-dependent manner. Scanning electron microscopic images revealed that drastic bacterial cell membrane damage with a rough surface and the deformation of cells was caused by the extract. Furthermore, confocal laser scanning microscopic (CLSM) images confirmed the inhibition of *S. algae* biofilm formation by RSC extract. RSC extract also suppressed bacterial motility, induced protein leakage, and reduced extracellular protease activity, thus highlighting its potent bactericidal effects. These findings suggested that the RSC extract rich in phenolic compounds could serve as an antimicrobial agent and hold promise as a natural preservative for perishable foods, especially seafoods.

## 1. Introduction

Seafood has gained increasing demand globally. Due to its high moisture content, nutrient-rich composition, and neutral pH, seafood is highly prone to spoilage, associated with rapid bacterial growth after death [1]. Spoilage contributes to undesirable flavors and loss in texture, thus causing rejection by consumers. Microbial metabolism plays a significant role in breaking down proteins in seafood during storage and processing, resulting in the formation of considerable undesirable products [2]. Microorganisms are able to produce large amounts of extracellular proteases, severely affecting the nutritional value of deteriorated products. Bacteria such as *Aeromonas* spp., *Pseudomonas* spp., *Serratia* spp. and *Shewanella* spp. exhibit high proteolytic activity on both myofibrillar and sarcoplasmic proteins in aquatic animals [3]. Among these microbes, *Shewanella* species, Gram-negative specific spoilage organisms (SSOs) commonly found in seafood, have been known as a major cause of spoilage in seafoods even at low temperatures and a small cell number can produce off-odors [4]. *Shewanella putrefaciens* and *Shewanella algae* are also the major spoilage flora and can degrade trimethylamine oxide (TMAO) into trimethylamine (TMA), generating off-flavor compounds like hydrogen sulfide, biogenic amines, and organic acids. As a result, the sensory and nutritional quality of aquatic products is detrimentally impacted by the proliferation of *Shewanella* species, in which the shelf life becomes shortened and the commercial value of the products is decreased [3,5].

To mitigate the aforementioned spoilage, chemical preservatives have been traditionally employed in the fish processing industry to retard the spoilage and retain freshness during storage [6]. However, the long-term use of synthetic chemicals for the shelf-life extension of seafood has raised health concerns [7]. Consequently, there is a need to discover safe, innovative natural antimicrobial agents that can help extend the storage duration of aquatic products. Natural substances have been employed as antimicrobial agents to provide several benefits beyond their preservative functions. In particular, natural substances from agricultural waste or by-products from the agriculture industry are a source of valuable natural substances. Over the past twenty years, global per capita nut consumption has significantly risen, owing to their role as functional foods [8]. However, by-products, like seed coat, which are often considered to have minimal market value, can be better exploited. The seed coats of various beans, including hazelnuts, cashew nut [9], and legumes, e.g., peanut and lentils [10,11,12], were rich in phenolic compounds, which offer a protective effect against oxidation and are linked to numerous physiological benefits, such as antioxidants, etc. [13,14]. Moreover, ethanolic extract from hickory nut’s seed coat had a high content of polyphenols and flavonoids and showed antibacterial activity against *Staphylococcus aureus* [15].

Bambara groundnut (BGN, *Vigna subterranean*) is mainly cultivated for its seed in the remote areas of sub-Saharan Africa [16]. It is also a native groundnut of the southern part of Thailand [17], consumed locally as a food source. The dehulling process is required before milling, while the seed coat, with low economic value, is generated. Generally, Bambara groundnut flour can be used as food. The seed coat can be used as an excellent source of active polyphenols to act as antimicrobial agents, etc. [17]. The seed coats of various Bambara groundnut varieties display a wide range of colors, which correspond to variations in their chemical compositions, and have potential linkage to the antimicrobial properties of the seed coat [18,19]. The most abundant pigments in the black, red, and dark brown pigments of the Bambara groundnut’s seed coat are anthocyanins and flavonoids, e.g., catechin, epigallocatechin, rutin, quercetin, kaempferol, etc. [18]. However, the antimicrobial properties of the seed coat (SC) of Bambara groundnuts having different colors have not been fully exploited. SC extract has been recognized for its antioxidant and antimicrobial properties without cytotoxicity [17]. Nevertheless, its application as an antimicrobial agent specifically against *Shewanella* species, along with its bactericidal effect and modes of action, has not yet been thoroughly elucidated. This study aimed to elucidate the effects of SC extract on *Shewanella* species inactivation by analyzing the time–kill profile, bacterial cell morphology, biofilm formation, and motility, as well as assessing cellular features like extracellular protease activity. In addition, the phenolic profiles of two SC extracts were analyzed.

## 2. Materials and Methods

### 2.1. Chemicals and Microbial Media

The chemicals (analytical grade) were purchased from Sigma-Aldrich Inc. (St. Louis, MO, USA), except for ethanol (98%) which was acquired from Merck (Darmstadt, Germany). Microbial media were purchased from OXOID^TM^, Thermo Fisher Scientific^TM^ (Waltham, MA, USA), and HiMedia Laboratories (Mumbai, India).

### 2.2. Preparation of Seed Coat

Two varieties of dried Bambara groundnut seed, Vigna subterranean (with red and white seed coats), were purchased from a supplier in Songkhla province and transported to a laboratory at the Prince of Songkla University, Thailand. The groundnut seeds were immersed in distilled water at 4 °C for 2 days (ratio 1:1, *w*/*v*). The red seed coat of Bambara groundnut (RSC) and white seed coat of Bambara groundnut (WSC) were peeled off, collected, washed three times with distilled water and dried (60 °C, 48 h) until a constant weight was gained. Then, the dried RSC and WSC were ground using a grinder and sieved through an 80-mesh sieve. The seed coat powders were kept in a vacuum bag at −20 °C until use.

### 2.3. Preparation of Seed Coat Extract

The ultrasound-assisted extraction was applied [19]. The 200 mL of 80% ethanol (*v*/*v*) was added to a 250 mL beaker containing 10 g of dried seed coat powder. Ultrasonic equipment (Sonics, Model VC750, Sonica & Materials, Inc., Newtown, CT, USA) with 70% sonication amplitude was applied for 40 min to the mixture placed in an ice bath. Thereafter, the seed coat pellet was removed by centrifuging at 5000× *g* (30 min, 4 °C). The supernatant was then filtered with Whatman #1 filter paper, and the solvent was removed from the filtrate using an Eyela rotary evaporator at 40 °C (Tokyo Rikakikai, Co. Ltd., Tokyo, Japan). Residual ethanol was removed by nitrogen purging. The aqueous solution was lyophilized and kept, in an amber bottle at −20 °C. The powders were analyzed.

### 2.4. Analyses

#### 2.4.1. Extraction Yield

The extraction yield was calculated from the weight of seed coat extract relative to that of dried seed coat powder. Yield in the percentage was recorded [20].

#### 2.4.2. Measurement of Total Phenolic Content (TPC)

The TPC of dried seed coat extract powder was examined using Folin–Ciocalteu’s reagent (FCR). Gallic acid was used as a standard phenolic compound in this study. The TPC was calculated and reported as mg gallic acid equivalent (GAE)/g dry extract [21].

#### 2.4.3. Analysis of Chemical Compounds Using Liquid Chromatography Quadrupole Time-of-Flight Mass Spectrometry (LC-Q-TOF/MS)

RSC and WSC extracts were dissolved in deionized water to obtain a concentration of 10 mg/mL for LC-Q-TOF/MS analysis. The LC-Q-TOF/MS profiling and identification of RSC and WSC extracts under the optimized conditions were performed using the LC-Q-TOF/MS machine Agilent G6545A (Agilent Technologies, Waldbronn, Germany) following the procedure of Boukaew et al. [22]. The sample was initially separated using an ultra-high-performance liquid chromatography (UHPLC) column (Zorbax Eclipse Plus C18 Rapid Resolution HD 150 mm length × 2.1 mm inner-diameter, particle size 1.8 μm, Agilent) at a temperature of 25 °C. Mobile phases A and B were 0.1% formic acid in water and 0.1% formic acid in ACN, respectively. The gradient program for the mobile phase was set following the procedure of Boukaew et al. [22] involving a flow rate of 0.2 mL/min and a temperature of 25 °C. The positive and negative modes were employed. Mass hunter METLIN metabolite PCD (Personal Compound Database) and PCDL (Personal Compound Database and Library) version 8 were adopted to analyze the compounds.

### 2.5. Determination of Antimicrobial Activity

#### 2.5.1. Bacterial Strains and Preparation

Two species of *Shewanella* were selected for testing due to their well-known spoilage microorganisms in seafood [5]. *S. putrefaciens* JCM 20190 was obtained from the Japan Collection of Microorganisms, while *S. algae* TBRC 5775 was acquired from Thailand’s National Center for Genetic Engineering and Biotechnology. Both strains were preserved in a 20% glycerol solution at −80 °C. The strain identification was confirmed through 16S rRNA gene sequencing. Before testing, the bacteria were cultured in tryptic soy broth (TSB) to obtain the log phase (~9 log CFU/mL). Bacterial concentration was measured by reading optical density at 600 nm (OD_600_) and comparing it to the 0.5 McFarland standard to achieve the bacterial concentration of 10^8^ CFU/mL. The bacterial inoculum was prepared with a suitable medium for each test to obtain the bacterial density of approximately 6 log CFU/mL.

#### 2.5.2. Determination of Minimum Inhibitory Concentration (MIC) and Minimum Bactericidal Concentration (MBC) of Seed Coat Extracts of Bambara Groundnut

The antimicrobial activity was assessed using the colorimetric microdilution method. Mueller–Hinton broth (MHB) was used to assess the MIC and MBC of RSC and WSC extracts in a 96-well microplate. Briefly, the extract (100 µL) was added to a sterile 96-well plate and diluted with MHB using a 10-fold serial dilution method to obtain various concentrations. Subsequently, 100 µL of bacterial suspension cultured in MHB at 1.5 × 10^6^ CFU/mL was added. The final concentrations of extract ranged from 0.0625 to 32 mg/mL. A bacterial suspension excluding the extracts was considered as a positive control, while a negative control was prepared using only media without bacterial suspension. Moreover, potassium sorbate at various concentrations with bacterial suspension was used as a standard food preservative control. The 96-well microplate was incubated at 37 °C for 15 h. The 10 µL of resazurin dye solution (0.18%, *w*/*v*) was added to each well and further incubated for 3–9 h. After incubation, the pink and blue colors of the solution in the well indicated bacterial growth and lack of growth, respectively. The resazurin is a blue color in an oxidized state and turns to a pink color (resorufin) when reduced by the viable bacteria in the solution. The lowest concentration of extract, which inhibited the growth of bacteria (blue color), was recorded as the MIC value. To validate the survival of bacteria, 10 µL of suspension was collected from wells, and dropped and spread on Mueller–Hinton agar. Incubation was further performed at 37 °C for 24 h. The MBC value was recorded by the lowest concentration showing a bactericidal effect (no growth).

#### 2.5.3. Time–Kill Kinetics of Seed Coat Extracts of Bambara Groundnut

The time–kill profiles of both RSC and WSC extracts against *S. putrefaciens* and *S. algae* were examined according to the procedure of Palamae et al. [23]. Bacterial inoculum of approximately 6 log CFU/mL in TSB was mixed with the RSC or WSC extracts at the final concentrations of MIC/4, MIC/2, MIC, 2MIC, and 4MIC. The untreated bacterial culture was used as a negative control. During incubation (37 °C, 24 h), bacterial suspensions were collected at 0, 2, 4, 6, 8, 10, 12, and 24 h. The bacterial suspensions were diluted using a serial dilution with 0.85% NaCl (*w*/*v*), and the viable bacterial cells were counted on TSA plates.

#### 2.5.4. Scanning Electron Microscopy (SEM)

SEM was employed to visualize the morphological changes of *S. putrefaciens* and *S. algae* under various treatments: untreated (control), 4MIC of SC extract treated, and potassium sorbate treated samples. The samples were prepared, and analysis was performed as detailed by Palamae et al. [23] using a scanning electron microscope (FEI Quanta 400-ESEM FEG, Hillsboro, OR, USA).

#### 2.5.5. Protein Leakages of *Shewanella* Species Cells Treated with Red Seed Coat Extract from Bambara Groundnut

*S. algae* cells in their log phase were suspended in PBS (1 × 10^6^ CFU/mL) containing the RSC extract at MIC/2, MIC, 2MIC, and 4MIC. The mixtures were incubated at 37 °C for 24 h, while untreated bacteria served as the negative control. After 24 h, the cell suspensions were collected, and filtration was done with the aid of a 0.22 µm Millipore filter. Protein leakage into the filtrates was measured using the Bradford assay [24], and the OD_595_ values were read with a FLUOstar Omega microplate reader (BMG Labtech GmbH, Ortenberg, Germany). A bovine serum albumin standard solution (1.95–15.60 µg/mL) was used to quantify the protein concentration.

#### 2.5.6. Confocal Laser Scanning Microscopy (CLSM) Analysis of Biofilms

*S. algae* had a strong biofilm formation (BFI) index, whereas *S. putrefaciens* had a weak BFI index. Therefore, *S. algae* was selected to determine the biofilm formation using CLSM analysis. The bactericidal actions of RSC extract on the biofilm formation of *S. algae* were visually confirmed by CLSM analysis, as described previously [3]. Briefly, *S. algae* cells (8 log CFU/mL) were treated with RSC extract (MIC/2, MIC, and 2MIC) and Luria Bertani Broth (HiMedia Laboratories, Mumbai, India) in a 24-well polystyrene cell culture plate (Corning, NY, USA). The 24-well plate was incubated (37 °C, 48 h) under the static condition. The biofilm supernatants were carefully removed. The wells were washed twice with 0.1 M phosphate buffer solution (PBS) (pH 7.2). Subsequently, the biofilm was stained with SYBR Green I (Sangon Biotech, Co., Ltd., Shanghai, China) under dark conditions for 30 min. CLSM imaging of biofilm formation was carried out using a Zeiss LSM 800 Airyscan confocal laser scanning microscope with ZEN 2.5 software (Carl Zeiss, Jena, Germany) at a 488 nm excitation light and 500–550 nm emitting light with a 20× microscope objective. Two- and three-dimensional biofilm structure and biofilm thickness were measured. The biofilm thickness analysis was conducted in triplicates by z-stack mode.

#### 2.5.7. Anti-Swimming and Swarming Motilities

The swimming and swarming capabilities of *S. algae* were assessed at 37 ± 2 °C [23]. RSC extract suspension was prepared in sterilized distilled water and filtered by a 0.20 µM syringe filter membrane. The RSC extract suspension was diluted with Luria Broth (LB) medium with 1.5% and 0.3% agar for swarming and swimming tests, respectively, in a sterile polystyrene 6-well plate (35 mm) to obtain the final concentrations of MIC, MIC/2, MIC/4, and MIC/8. The well without the RSC extract was considered as the control. The swimming test was done using the overnight culture by transferring the culture with a sterilized needle into the center of the well. The diameter of bacterial growth was measured after incubation for 2, 4, 6, and 8 h. For the swarming test, overnight culture (2 µL) was added to the center of each well. The diameter of bacterial growth was examined after 6, 18, and 24 h.

#### 2.5.8. Extracellular Protease Activity

Extracellular protease activity was evaluated using the modified Oxford Cup method [3]. Briefly, the agar plate was prepared with 1.5% (*w*/*v*) agar and 1% (*w*/*v*) skim milk, and then holes were punched for 4 wells/plate with a sterilized 1000 µL pipette tip. One–hundred μL of the log phase bacterial solution (~10^8^ CFU/mL) in 0.85% NaCl (*w*/*v*) was transferred to the well (diameter = 8 mm). Plates were incubated at 37 °C for 24 h, 48, and 72 h. Extracellular protease activity was evaluated in triplicate, in which clear zones (decomposition zone) surrounding the well were determined.

### 2.6. Statistical Analysis

All experiments followed a completely randomized design (CRD). Each treatment and analysis were run in triplicate. One-way analysis of variance (ANOVA) was used, followed by Tukey’s test for mean comparison. Significance was determined at a threshold of *p* < 0.05.

## 3. Results and Discussion

### 3.1. Extraction Yield, Total Phenolic Content, and Antimicrobial Activity of Seed Coat Extracts from Bambara Groundnut Prepared Using Ultrasound-Assisted Extraction Process

The extraction yield represents the efficiency of the ultrasound-assisted extraction (UAE) process for the extraction of active compounds in seed coats using ethanol as the medium. The RSC extract had a yield of 8.35%, whereas the WSC extract exhibited a much lower yield (2.34%) (*p* < 0.05). This discrepancy in yields suggested that the red seed coat rendered more extractable compounds when subjected to the UAE process, likely due to differences in the compositions and structural properties of both seed coats [17].

Phenolic compounds are known for their antimicrobial properties and contribute to numerous health benefits. The RSC extract showed a TPC of 420.98 ± 0.27 mg of gallic acid (GAE)/g dry extract. The WSC extract had a TPC of 28.29 ± 0.91 mg GAE/g dry extract. This substantial difference indicated that the red seed coat was richer in phenolic compounds than the white counterpart. The higher TPC and extraction yield of RSC extract implied that the extract from RSC contained a larger amount of antibacterial bioactive compounds, particularly phenolics, than the extract from the white seed counterpart. These findings might stem from inherent variations in the seed coat composition, where the red pigmentation could be associated with a greater presence of phenolic substances. For lentil seeds, several phenolic compounds, primarily found in the seed coat, were linked to different background colors [12]. Pathiraja et al. [12] studied lentil (*Lens culinaris*) seed coats having various colors and found significant variation in TPC, which ranged from 15.2 µg/g in grey-colored seeds (low tannin) to 65.0 μg/g in green-colored seeds (high tannin). A previous study reported that Bambara seeds had a relatively low total phenolic content (TPC), ranging from 0.75 to 17.71 mg GAE/g flour [19]. The lower TPC was due to the presence of flour and other components, compared to the TPC found in only the seed coat of Bambara groundnuts, ranging from 169.2 to 569.2 mg GAE/g dry extract [17]. The average TPC followed a descending order based on seed coat color across all landraces: black > red > brown > cream > white [19]. Typically, the TPC in legume seeds is influenced by the seed coat’s color, in which the darker seeds had higher TPC and lighter seeds showed lower levels [19].

The antimicrobial activity of the plant extract was closely associated with its concentration. The phenolic compounds in the extracts are primarily responsible for their antimicrobial properties [17]. The antimicrobial activity of RSC and WSC extracts, which contained varying TPCs, was evaluated against Gram-negative bacteria, *S. putrefaciens* and *S. algae*, as shown in Table 1. The RSC extract demonstrated stronger inhibition, with MIC and MBC values of 4 and 8 mg/mL against *S. putrefaciens*, and 8 and 32 mg/mL against *S. algae*, respectively. In comparison, the WSC extract exhibited weaker antimicrobial activity, with MIC and MBC values exceeding 32 mg/mL for both bacterial strains. Potassium sorbate, a common food preservative, exhibited moderate inhibition against *S. algae* (MIC of 16 mg/mL, MBC of 64 mg/mL), while showing stronger activity toward *S. putrefaciens* (MIC of 4 mg/mL, MBC of 8 mg/mL). Overall, RSC extract was more effective than WSC extract in inhibiting both *S. putrefaciens* and *S. algae*, with efficacy comparable to that of potassium sorbate, particularly against *S. putrefaciens*. It is widely known that Gram-negative bacteria are generally more resistant to plant extracts because of the presence of lipopolysaccharides in their outer membranes [18]. However, Bambara groundnut (BGN) extracts from red and brown hulls demonstrated a greater inhibition of Gram-negative bacteria, such as *Klebsiella pneumoniae* ATCC 700603 and *Pseudomonas aeruginosa* ATCC 27853, compared to Gram-positive bacteria like *S. aureus* ATCC 33591 [25]. The antimicrobial potential of BGN extracts was largely associated with the high phenolic content, particularly flavonoids and tannins, found in red and brown hull varieties [18]. In a study by Klompong and Benjakul [8], SC extracts inhibited the growth of bacteria such as *S. aureus*, *Escherichia coli*, and *Bacillus cereus* in a dose-dependent fashion using the agar diffusion procedure. The general mechanism of action of these antimicrobial agents involves the disruption of the cytoplasmic membrane, leading to altered membrane permeability. These resulted in cytoplasm leakage, coagulation, and ultimately the deformation, lysis, and death of the bacterial cells [26,27].

### 3.2. Chemical Compositions of Seed Coat Extract from Bambara Groundnut Analyzed by Liquid Chromatography Quadrupole Time-of-Flight Mass Spectrometry (LC-Q-TOF/MS)

The compounds extracted from both RSC and WSC extracts analyzed using LC-Q-TOF/MS in negative mode are shown in Table 2. The dominant compounds of RSC extract were flavonoids, in which quercetin 3-galactoside (9.045 × 10^6^ abundance) and rutin (6.252 × 10^6^ abundance) were the most abundant compounds. A variety of other flavonoids, polyphenols, and phenols were also identified at the lower abundances. Meanwhile, the dominant compounds of WSC extract were triterpenoid saponins, especially calendasaponin B (6.580 × 10^6^ abundance). Other saponins, sesquiterpenoids, and benzoates were also detected in notable amounts (Table 2).

Based on the positive mode, RSC extract contained terpenoids, e.g., Foeniculoside VII (10.224 × 10^6^ abundance) was the dominant compound, while flavonoids and other organic compounds appeared at lower abundances (Table 3). For the WSC extract, similar to the negative mode result, this extract showed a high abundance of terpenoids and saponins, such as Foeniculoside VII and Soyasapogenol B. Foeniculoside VII was again the most abundant compound, but with a slightly lower abundance, compared to RSC extract. The RSC extract appeared to be richer in flavonoids and polyphenols, known for their antioxidant properties. In contrast, WSC contained more triterpenoid saponins, which might have different bioactive properties, such as anti-inflammatory or anticancer effects [18,28]. These findings highlight the potential for each seed coat extract to serve distinct nutritional or medicinal purposes, depending on the bioactive compounds present.

### 3.3. Time–Kill Kinetics of Red Seed Coat Extracts from Bambara Groundnut Against Shewanella Species

**Figure 1:**
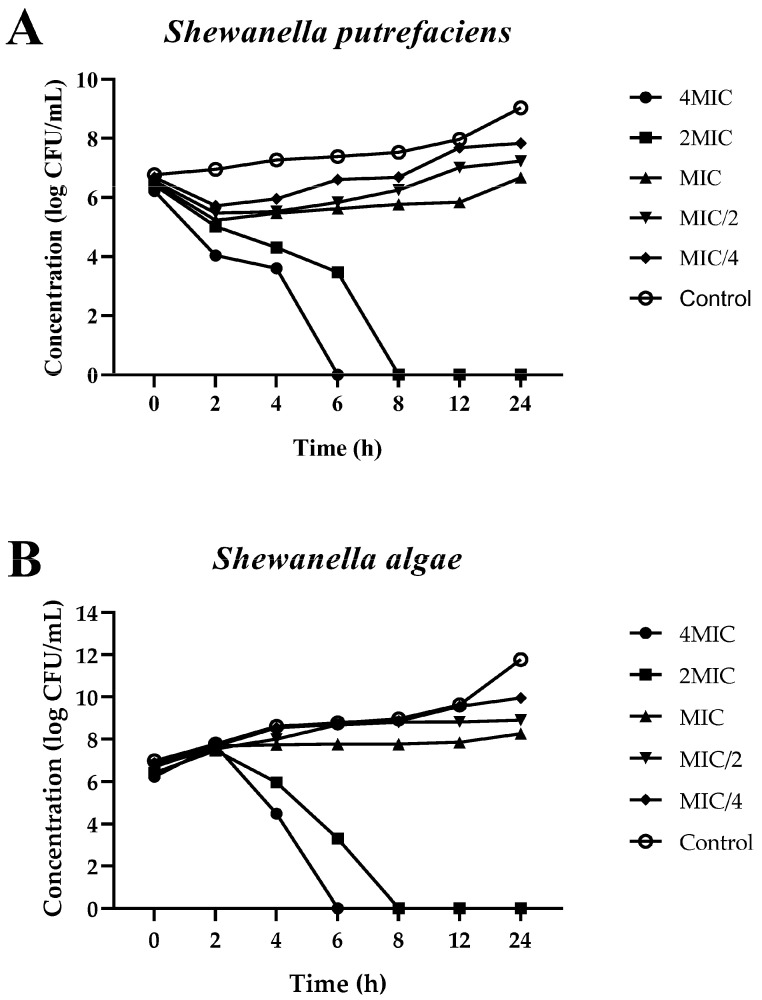
Time–kill curve of *Shewanella putrefaciens* (**A**) and *Shewanella algae* (**B**) after treatment with Bambara groundnut red seed coat extract (RSC extract) at different concentrations. The results are presented as means ± SD (*n* = 3).

The RSC extract exhibited a dose-dependent impact on the bacterial counts of *S. putrefaciens* and *S. algae* as shown in Figure 1. The most significant bacterial reduction occurred at concentrations above 2MIC, starting at 4 h of incubation for both species. In Figure 1A,B, a gradual decrease in bacterial concentrations was observed as a function of time. Notably, at higher concentrations (2MIC and 4MIC), the reduction in bacterial load was more pronounced with a non-detectable count by 8 and 6 h, respectively. In contrast, lower concentrations (MIC/2 and MIC/4) displayed moderate antibacterial activity. The RSC extract plausibly disrupted bacterial cell replication, as evidenced by the sharp decline in microbial load over time. In contrast, no reduction in bacterial count was observed in the control group. The RSC extract exhibited strong antibacterial activity toward both *S. putrefaciens* and *S. algae*, particularly at higher concentrations. Its ability to decrease bacterial load in a dose- and time-dependent manner highlighted its potential as a natural antimicrobial agent.

### 3.4. Effect of Red Seed Coat Extract from Bambara Groundnut on Morphology of Shewanella Species Cells

**Figure 2:**
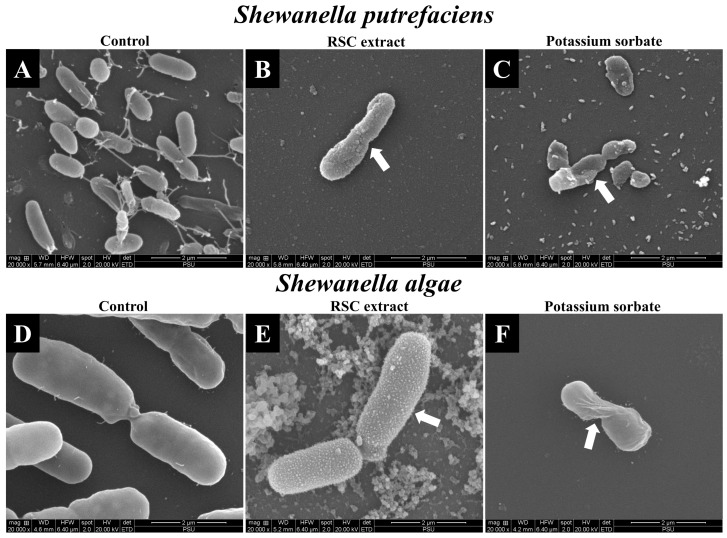
Scanning electron microscopy of *Shewanella putrefaciens* (**A**–**C**) and *Shewanella algae* (**D**–**F**); untreated cells (**A**,**D**); cells treated with 4MIC of Bambara groundnut red seed coat extract (RSC extract) (**B**,**E**); and cells treated with 4MIC of potassium sorbate (**C**,**F**). The magnification is ×20,000 and the scale bar is 2 µm. The white arrow indicates damaged bacterial cells.

The impact of RSC extract and potassium sorbate on the morphology of *Shewanella* species, observed through scanning electron microscopy (×20,000) is illustrated for *S. putrefaciens* in Figure 2A–C and *S. algae* in Figure 2D–F. Untreated *S. putrefaciens* and *S. algae* cells exhibited smooth surfaces and rod-shaped structures, as seen in Figure 2A,D. Notably, *S. algae* cells were larger, compared to those of *S. putrefaciens*. *Shewanella* species generally exhibit a rod-shaped morphology and have a size range of 1–3 µm [29], whereas *S. putrefaciens* cells tend to be smaller, typically between 0.5 and 2.0 µm [30]. However, treatment with the RSC extract caused significant damage to *Shewanella* species. After exposure to RSC extract at 4MIC, *S. putrefaciens* cells exhibited morphological changes such as surface roughness, distortion, and size elongation, while *S. algae* cells displayed rough surfaces and an accumulated extract was found on the cell surface (Figure 2B,E). These alterations impaired essential functions of cells such as cell division, repair, and metabolism, ultimately leading to reduced antibacterial resistance and cell death. *S. putrefaciens* and *S. algae* cells treated with potassium sorbate (Figure 2C,F) showed similar distortions, including rough or shrunken surfaces, size reduction, degenerative changes, and cell membrane damage. Consistent with earlier research, Klompong and Benjakul [8] documented that SC extract (5 mg/mL) caused significant damage to the cells of *S. aureus*, *E. coli*, and *B. cereus*. SEM photomicrographs revealed cell shrinkage, deformation, and rupture.

### 3.5. Changes in Biofilms as Affected by Red Seed Coat Extract from Bambara Groundnut

**Figure 3:**
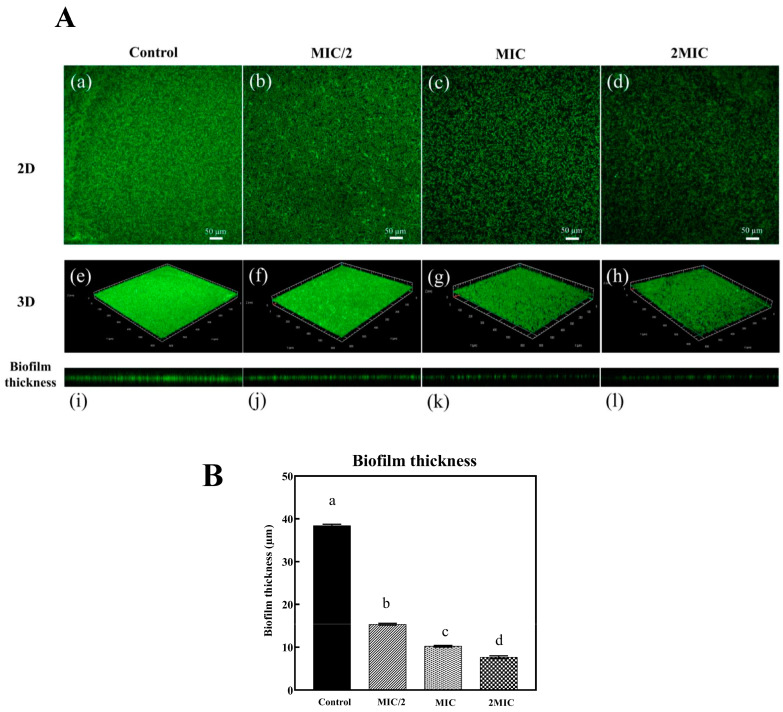
(**A**) CLSM images for 2D (a–d), 3D (e–h), and z-stack (i–l) images (biofilm thickness) of live cells of *Shewanella algae* developed on a 24-well plate treated with Bambara groundnut red seed coat extract (RSC extract) at different concentrations (scale bar: 50 µm). (**B**) Effect of RSC extract at different concentrations on biofilm thickness. Different lowercase letters within the same MIC on the bars denote a significant difference (*p* < 0.05). The results are presented as means ± SD (*n* = 3). Bars represent the standard deviation (*n* = 3).

*S. algae* has been known to possess various virulence factors, including siderophores, tetrodotoxin, and pufferfish toxin [31]. It was hypothesized that β-hemolysins, associated with hemolytic activity, contributed to the higher virulence of *S. algae* compared to *S. putrefaciens* [29,32]. Additionally, antimicrobial tests in this study revealed that *S. algae* showed greater resistance to RSC extract than *S. putrefaciens*. Additionally, *S. algae* can form biofilm in the well of a 24-well plate with a strong BFI index. As a result, *S. algae* was selected for CLSM investigation instead of *S. putrefaciens*. Living bacteria under biofilm through 2D and 3D CLSM images are shown in Figure 3A. The results were validated by the presence of live bacterial biofilm populations in 24-well plates, as determined by a live viability assay kit containing SYBR Green I. In Figure 3A(a,e), the control group (untreated *S. algae* cells) displayed dense, green-fluorescent bacterial biofilms. In contrast, a dose-dependent reduction in the viable biofilm and bacterial cell populations was observed in the sample treated with SC extract at concentrations of MIC/2 [Figure 3A(b,f)], MIC [Figure 3A(c,g)], and 2MIC [Figure 3A(d,h)]. This reduction was evidenced by the decreased number of live, green-stained cells. The CLSM images, therefore, confirmed the antibiofilm activity of the RSC extract, which correlated well with its bactericidal effect. This eventually led to the inhibition of *S. algae* biofilm formation related to a reduction in viable cells. The biofilm thickness in the presence of RSC extract at various concentrations (control, MIC/2, MIC, and 2MIC) is illustrated in Figure 3A(e–h),B. A marked reduction in biofilm thickness was observed in the RSC-treated groups, with decreases of 80%, 73.3%, and 60% for 2MIC, MIC, and MIC/2, respectively (*p* < 0.05), compared to the control group. These findings suggested that RSC extract at higher concentrations (MIC and 2MIC) was more effective in reducing biofilm formation. Quercetin 3-galactoside, a major component in RSC extract, has been found to be a major compound in the leaf extract of *Paederia foetida* Linn. The *P. foetida*’s leaf extract has shown a significant reduction in biofilm formation at MIC/2, MIC/4, and MIC/8 concentrations. Moreover, *P. foetida*’s leaf extract (MIC/2) has affected the downregulation of the *sarA* gene related to biofilm formation. Based on in silico molecular docking analysis, quercetin 3-galactoside has a particular binding to the SarA protein (regulated protein for biofilm formation) of *S. aureus* [33]. Rutin, a major component in RSC extract, has been reported for antibacterial activity (MIC = 200 µg/mL) and antibiofilm formation inhibition of *P. aeruginosa* [34]. Overall, the bactericidal action of RSC extract was also correlated with its ability to disrupt biofilm formation via interfering with the biofilm matrix. This phenomenon limited bacterial adhesion and growth.

### 3.6. Protein Leakage as Affected by Red Seed Coat Extract from Bambara Groundnut

**Figure 4:**
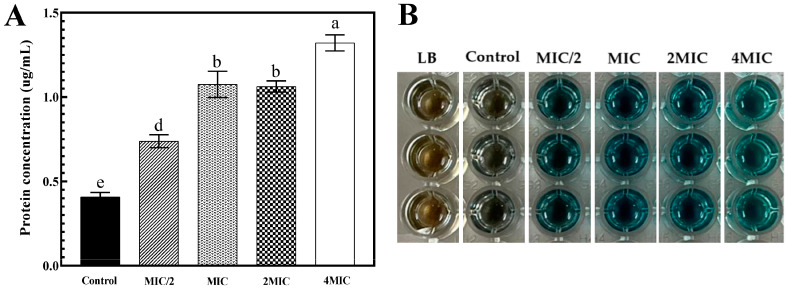
Quantitative (**A**) and qualitative (**B**) analyses of protein leakage of *Shewanella algae* treated with Bambara groundnut red seed coat extract (RSC extract) at different concentrations for 24 h. Different lowercase letters within the same MIC on the bars denote a significant difference (*p* < 0.05). The results are presented as means ± SD (*n* = 3). Bars represent the standard deviation (*n* = 3). The blue color indicates the presence of protein when tested using the Bradford assay.

The bacterial membrane is a crucial structure that protects cells from damage caused by antibiotics, disinfectants, preservatives, and antimicrobial agents [35]. Protein leakage from *S. algae* cells treated with RSC extract at varying concentrations for 24 h was observed in Figure 4A,B. The amount of protein leaked from *S. algae* treated with the RSC extract at MIC and 2MIC showed no significant difference (*p* > 0.05), but both levels showed higher protein leakage than that observed in the MIC/2 treated and control groups (Figure 4A) (*p* < 0.05). Moreover, in cells treated with 4MIC, the protein leakage reached approximately 1.32 µg/mL. The highest protein leakage, about three-times higher than the control, was recorded in the 4MIC-treated group. This suggested that the RSC extract significantly affected the outer surface of Gram-negative bacteria, causing protein leakage and potentially releasing genetic material or other cellular components.

### 3.7. Inhibition of Swimming and Swarming Motilities by Red Seed Coat Extract from Bambara Groundnut

**Figure 5:**
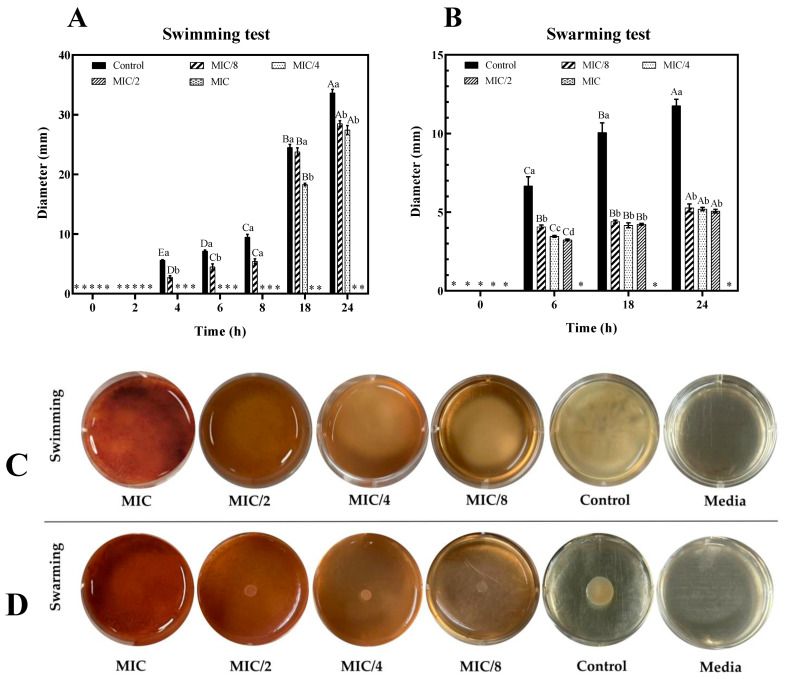
Effect of Bambara groundnut red seed coat extract (RSC extract) at different concentrations on swimming (**A**,**C**) and swarming (**B**,**D**) motilities. Images of swimming and swarming at 24 h with RSC extract at different concentrations. Different uppercase letters within the same MIC on the bars denote a significant difference (*p* < 0.05). Different lowercase letters within the same time on the bars denote a significant difference (*p* < 0.05). Different colors of media in each well are effected by the color of the RSC extract. The results are presented as means ± SD (*n* = 3). Bars represent the standard deviation (*n* = 3). * Means no extracellular protease activity.

Bacteria can adhere to surfaces and colonize host cells through directional motility, which is facilitated by flagella. Swimming and swarming motility are important for the properties of bacteria to survive in unnatural conditions. Swimming motility is the movement of bacteria in a liquid or low-viscosity medium using flagella rotation, and swarming motility is the movement over the surface of a semisolid medium using flagella rotation. Therefore, the inhibition of motility can also inhibit the activity of putrefactive bacteria. In the control group of untreated *S. algae*, a faster rate of movement was observed in the swimming assay compared to the swarming assay. Similar observations were reported by Li et al. [11], where *S. putrefaciens* BNCC 337021 demonstrated greater swimming ability than swarming ability. As shown in Figure 5A–D, the diameter of the bacterial halo decreased as the concentration of RSC extract increased, and both motility types of *S. algae* were progressively restricted at concentrations greater than MIC/2 (*p* < 0.05). The inhibition of swimming and swarming motility could be primarily linked to the expression of genes related to flagella [36]. Their findings suggested that the extract disrupted the flagellar system, leading to disorganized flagellar assembly. In fact, swimming and swarming halos were barely visible at MIC/2 and MIC concentrations, respectively, even after 24 h of incubation. Notably, swimming motility was inhibited by the RSC extract more than swarming motility. According to Nassar et al. [15], swimming motility involves the movement of individual cells, whereas swarming motility is a collective movement of bacterial populations. Thus, both the individual cell movement and collective population movement of *S. algae* were more inhibited when the RSC extract was applied at concentrations higher than the MIC. As previously documented, both motility mechanisms are closely linked to bacterial flagella and cell surface characteristics. RSC extract might negatively affect the synthesis and development of *S. algae* flagella, thereby lowering its motility.

### 3.8. Extracellular Protease Activity as Affected by Red Seed Coat Extract from Bambara Groundnut

**Figure 6:**
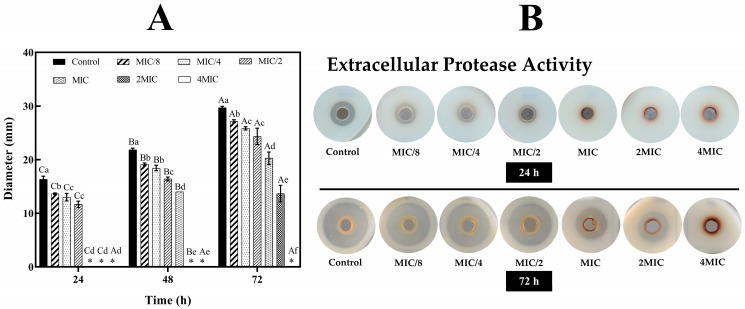
Effect of Bambara groundnut red seed coat extract (RSC extract) at different concentrations on (**A**) the diameter of the extracellular protease clear zone of *Shewanella* algae after incubation at 37 °C for 24 h, 48 h and 72 h. (**B**) Images of the extracellular protease clear zone at 24 h and 72 h, respectively, treated with RSC extract at different concentrations. Different uppercase letters within the same MIC on the bars denote a significant difference (*p* < 0.05). Different lowercase letters within the same time on the bars denote a significant difference (*p* < 0.05). The results are presented as means ± SD (*n* = 3). Bars represent the standard deviation (*n* = 3). * Means no motility.

Extracellular proteases are known to break down external substrates, leading to significant protein damage [37]. The extracellular protease activity of *S. algae* was evaluated in the presence of RSC extract, as depicted in Figure 6. In the agar plate assay, a decomposition zone with a diameter of 16.3 mm was observed after 24 h in the untreated *S. algae* (control). No such zone was observed at MIC, 2MIC, and 4MIC concentrations. After 72 h of incubation, the decomposition zones for untreated *S. algae*, MIC/8, MIC/4, MIC/2, MIC, and 2MIC were 29.7, 27.2, 25.8, 24.3, 20.3, and 13.7 mm, respectively (*p* < 0.05) (Figure 6A). This suggested that RSC extract at higher concentrations effectively suppressed the degradation process of extracellular proteases [3]. No decomposition zone was present at 4MIC, indicating a strong inhibitory effect on extracellular proteases (Figure 6B). This inhibition was likely responsible for the reduction in cell metabolism, which correlated with decreased proteolytic activity. For *S. putrefaciens* BNCC 337021, a common spoilage organism in marine fish, the cellular metabolism was significantly reduced by the combination of CO_2_ and low temperature, as shown by the reduced extracellular protease activity [3]. Additionally, Wang et al. [38] documented that the extracellular protease activity of *Pseudomonas fragi* was reduced when exposed to harsh conditions.

## 4. Conclusions

The red seed coat from Bambara groundnut (RSC extract) exhibited significantly higher extraction yield, total phenolic content, and antimicrobial activity compared to the white seed coat extract (WSC extract). RSC extract showed a potent inhibition of *S. putrefaciens* and *S. algae*, plausibly due to bioactive compounds in the extract, particularly flavonoids. The major flavonoid compounds in RSC extract, such as quercetin 3-galactoside, and rutin contribute to the antibacterial effect. Several studies have shown that quercetin 3-galactoside and rutin may have a combined effect on bacterial cells. The WSC extract contained fewer antibacterial substances, such as saponins and triterpenoids, as major components. Therefore, the antibacterial activity of the RSC extract was shown using time–kill kinetics, CLSM, and SEM analysis to confirm the bactericidal effect of RSC extract, which disrupted bacterial morphology and biofilm formation. Additionally, the RSC extract affected bacterial motility and extracellular protease activity, and caused protein leakage. These findings highlight the potential of RSC extract as a natural additive for the preservation of perishable seafoods and other products.

## Figures and Tables

**Table 1:** Antimicrobial activity of Bambara groundnut red seed coat extract (RSC extract) and Bambara groundnut white seed coat extract (WSC extract) against *Shewanella* species.

Extracts/Food Preservative	*Shewanella putrefaciens*		*Shewanella algae*	
	MIC (mg/mL)	MBC (mg/mL)	MIC (mg/mL)	MBC (mg/mL)
RSC extract	4	8	8	32
WSC extract	>32	>32	>32	>32
Potassium sorbate	4	8	16	64

MIC: minimum inhibitory concentration; MBC: minimum bactericidal concentration.

**Table 2:** Chemical compounds in the red and white seed coat of Bambara groundnut extracts analyzed by liquid chromatography quadrupole time-of-flight mass spectrometry (LC-Q-TOF/MS) in negative mode.

Identified Compounds	Compound Types	Formula	Mass	Rt (min)	Abundance (×10^6^)
Bambara groundnut red seed coat extract (RSC extract)					
Quercetin 3-galactoside	Flavonoid	C_21_H_20_O_12_	464.095	13.009	9.045856
Rutin	Flavonoid	C_27_H_30_O_16_	610.153	11.919	6.252689
6,8-Di-C-glucopyranosyltricetin	Flavonoid	C_27_H_30_O_17_	626.148	11.117	2.112860
Telephioidin	Flavonoid	C_21_H_20_O_13_	480.090	12.269	2.111958
Isoscoparin 2″-O-glucoside	Flavonoid	C_28_H_32_O_16_	624.168	12.357	1.332209
Quercetin 3-glucosyl-(1->2)-[rhamnosyl-(1->6)-galactoside]	Flavonoid	C_33_H_40_O_21_	772.205	11.330	1.312073
Procyanidin B2	Polyphenol	C_30_H_26_O_12_	578.142	9.651	1.296922
2,4-Dihydroxybenzoic acid	Polyphenol	C_7_H_6_O_4_	154.026	4.991	1.117104
Quercetin 3-methyl ether 3′-xyloside	Flavonoids	C_21_H_20_O_11_	448.100	15.013	1.084713
3,4-Dihydroxybenzaldehyde	Phenols	C_7_H_6_O_3_	138.031	9.325	0.915070
Bambara groundnut white seed coat extract (WSC extract)					
Calendasaponin B	Triterpenoid saponin	C_48_H_76_O_20_	972.493	18.929	6.580920
Cinncassiol C3	Sesquiterpenoid	C_20_H_30_O_7_	382.199	12.115	4.644700
Momordin IIa	Saponin	C_48_H_76_O_18_	940.503	19.129	3.632120
Elatoside H	Saponin	C_42_H_66_O_15_	810.440	19.931	3.256450
2,4-Dihydroxybenzoic acid	Benzoate	C_7_H_6_O_4_	154.027	4.899	2.697640
Piperonyl sulfoxide	Benzene derivative	C_18_H_28_O_3_S	324.175	37.782	2.364110
Soyasaponin bg	Triterpenoid saponin	C_54_H_84_O_21_	1068.550	20.846	1.708810
Vinaginsenoside R12	Triterpenoid	C_36_H_64_O_11_	672.446	29.452	1.610460
S-Japonin	Sesquiterpenoid	C_19_H_28_O_3_S	336.176	35.716	1.487800
Methyl 2,4,6-trihydroxybenzoate	Phenol	C_8_H_8_O_5_	184.037	2.844	0.985210

**Table 3:** Chemical compounds in Bambara groundnut red and white seed coat extracts were analyzed by liquid chromatography quadrupole time-of-flight mass spectrometry (LC-Q-TOF/MS) in positive mode.

Identified Compounds	Compound Types	Formula	Mass	Rt (min)	Abundance (×10^6^)
Bambara groundnut red seed coat extract (RSC extract)					
Foeniculoside VII	Terpenoid	C_16_H_28_O_8_	348.179	10.401	10.224960
Luteolin 6-C-glucoside 8-C-arabinoside	Flavonoid	C_27_H_30_O_16_	610.155	11.741	3.318217
Cardiogenol C	Aromatic amine	C_13_H_16_N_4_O_2_	260.127	8.309	1.530854
(−)-Euphomine	Terpenoid	C_32_H_44_O_8_	556.306	29.091	1.175419
7-aminoflunitrazepam	Benzodiazepine	C_16_H_14_FN_3_O	283.111	29.543	1.061559
Betavulgarin glucoside	Isoflavone	C_23_H_22_O_11_	474.116	8.146	0.717766
Varenicline	Organic amino compound	C_13_H_13_N_3_	211.111	14.410	0.562498
Boviquinone 4	Terpenoid	C_26_H_36_O_4_	412.262	37.873	0.506933
Bambara groundnut white seed coat extract (WSC extract)					
Foeniculoside VII	Terpenoid	C_16_H_28_O_8_	348.180	10.412	7.438520
Soyasapogenol B 3-O-[a-L-rhamnosyl-(1->4)-b-D-galactosyl-(1->4)-b-D-glucuronide]	Triterpene saponin	C_48_H_78_O_18_	942.519	19.406	6.134970
24-Methylcycloart-23-en-3beta-yl acetate	Terpenoid	C_33_H_54_O_2_	482.411	36.368	1.783770
7-aminoflunitrazepam	Benzodiazepine	C_16_H_14_FN_3_O	283.111	29.052	1.517290
Calendasaponin B	Terpenoid	C_48_H_76_O_20_	972.491	19.005	0.976930
[12]-Gingerdione	Phenol	C_23_H_36_O_4_	376.262	31.958	0.939150
Cardiogenol C	Aromatic amine	C_13_H_16_N_4_O_2_	260.128	8.257	0.753460
N-butyryl-L-Homocysteine thiolactone	Amide	C_8_H_13_NO_2_S	187.067	8.420	0.73782

## Data Availability

The original contributions presented in the study are included in the article, further inquiries can be directed to the corresponding author.
